# The Importance of a Continuously Changing Heart Rate in Venous and Arterial Pressure Analysis

**DOI:** 10.18103/mra.v13i8.6824

**Published:** 2025-08-25

**Authors:** Gabriel P. Bonvillain, Lauren D. Pierce, Adria Abella Villafranca, Sam E. Stephens, Luke E. Ferguson, Hanna K. Jensen, Joseph A. Sanford, Jingxian Wu, Kevin Sexton, Morten Jensen

**Affiliations:** 1 Department of Biomedical Engineering, University of Arkansas, Fayetteville, Arkansas; 2 Department of Biomedical Informatics, University of Arkansas for Medical Sciences, Little Rock, Arkansas; 3 Department of Surgery, University of Arkansas for Medical Sciences, Little Rock, Arkansas; 4 Department of Anesthesiology, University of Arkansas for Medical Sciences, Little Rock, Arkansas; 5 Department of Electrical Engineering, University of Arkansas, Fayetteville, Arkansas

## Abstract

**Purpose:**

Previous studies have suggested that minimally invasive peripheral venous and arterial pressure waveforms provide a greater ability to detect acute changes in blood volume than traditional vital signs. Many of these studies are using Fast Fourier Transforms and power spectral densities to evaluate changes in the power at the heart rate frequency. Using the frequency domain requires a segment of the time domain to be converted into the frequency domain, which means that the heart rate derived from frequency domain analysis is an average of the segment used. However, in clinical settings the heart rate is changing continuously.

**Methods:**

This study evaluates the changing heart rate frequency power under varying time segments and compares the heart rate obtained from Fast Fourier Transform and power spectral density analysis with the instantaneous heart rate to gain a better insight into how a changing heart rate may influence the heart rate frequency power.

**Results:**

Spectral analysis revealed non-linear trends in heart rate frequency power, with changes that correspond to changes in the heart rate. We found the time segment chosen and the absolute difference between the instantaneous heart rate and the average heart rate obtained from power spectral density analysis influences the heart rate frequency power, such that as the instantaneous heart rate approaches the average heart rate, the heart rate frequency power increases.

**Conclusion:**

These results suggest that the time segment chosen for frequency domain analysis influences the power spectrum of the pressure waveforms. Furthermore, this study emphasizes the importance of utilizing a continuously changing heart rate in pressure waveform analysis.

## Introduction

Traumatic injury accounts for a considerable number of hospitalizations worldwide, and of these hospitalizations, hemorrhage remains one of the leading causes of preventable death ^1,2^. Currently, traditional vital signs such as blood pressure and heart rate are insufficient methods for detecting acute hemorrhage ^3,4^. This has led researchers to investigate the use of peripheral venous and peripheral arterial pressure waveforms to provide a more reliable detection of hemorrhage.

The arterial pressure waveform was initially identified as a potential signal for detection of acute hemorrhage due to the waveforms ability to identify rapid changes in systolic, diastolic, and mean arterial pressures ^5^; however, acquisition of the central arterial pressure waveform relies on invasive central arterial catheterization, so it is most often used in critically ill or anesthetized perioperative patients ^6^. Recently, studies have begun to identify changes in pulse wave velocity and pulse wave reflection in the peripheral arterial pressure waveforms that are associated with arterial stiffness and hemorrhage induced vasoconstriction ^7,8^. Procedures conducted to obtain peripheral arterial pressure waveforms are considerably less invasive than obtaining central arterial pressure waveforms from central arterial catheterization ^9,10^. Additionally, frequency domain metrics have been proposed as a method for interpreting the heart rate, harmonics, and primary and reflected waves ^8,11^.

The venous system is largely compliant and contains the primary blood reservoirs in the body, therefore, the peripheral venous pressure waveform was identified as a potential signal for detecting changes in blood volume ^12,13^. Due to the relatively low strength of the peripheral venous signal, the utility and characterization of this signal was limited until recent amplification methods were identified that allow for detection of subtle changes in the waveform ^14,15^. Previously, studies have utilized the Fast Fourier Transform (FFT) and power spectral densities (PSD) to identify changes in respiratory and cardiac frequency modulations and demonstrated that peripheral venous pressure waveforms are more sensitive to detecting changes in blood volume than traditional vital signs ^16–18^.

Additionally, studies have proposed hydromechanical interactions between the peripheral arterial and venous pressure waveforms that influence the FFT of each of these signals ^17,19,20^. Using a FFT requires a segment of a time domain signal that is converted to a frequency domain signal. Therefore, the heart rate frequency obtained by FFT is an average of the segment used; however, in clinical settings the heart rate is changing continuously. As researchers are proposing to use these metrics as a method for detecting changes in blood volume, one must consider the utility of these methods within physiological parameters. To the authors’ best understanding, there are currently no studies depicting how a changing heart rate may influence the cardiac frequencies obtained using an FFT. Because of the proposed correlations, this study evaluates how a continuously changing heart rate may influence the heart rate frequency obtained from peripheral venous and arterial pressure FFT’s.

## Methods

### DATA ACQUISITION

In a controlled study conducted in accordance with the internal review board at the University of Arkansas for Medical Sciences (UAMS), data was obtained from four female porcine subjects with an average weight and age of 72. 8 ± 1.6 kg and 16. 8 ± 0.4 weeks, respectively.

Each subject was induced under a general anesthetic consisting of propofol at a loading concentration of 0.5 mg/kg/min and isoflurane at a minimum alveolar concentration (MAC) of 1.5%. Then, the respiratory rate was fixed at twelve beats per minute (0.2 Hz) by mechanical ventilation using a Datex Ohmeda Aestiva 5 anesthesia machine (*GE Healthcare, Wauwatosa, Wisconsin, USA*).

### PERIPHERAL VENOUS PRESSURE WAVEFORM

The peripheral venous pressure waveform was obtained from SPR-350S Millar Mikro-Tip Pressure Catheters (*Millar, Inc*., Houston, Texas, USA) for Pig 1 and Pig 2; however, after conducting two experiments, the catheter was switched to SPR-320 Millar Mikro-Tip Pressure Catheters for their ability to navigate through the narrow peripheral veins easier than the previously used Millar Catheters. During the data collection for Pig 1, the catheter was inserted directly into the Internal Jugular; however, for Pig 2, 3, and 4 the catheters were inserted into the extremities of the right arm of each pig. For every case, the catheter was advanced to the inferior aspect of the superior vena cava. The placement of the catheters was verified using a BK5000 Ultrasound system (*BK Medical, Herlev, Denmark*).

Data was obtained from the venous catheter by connecting the catheter to a Millar PCU-2000 Pressure Control Unit (*Millar Inc*.) by a pair of PED-10D cables. An NI USB-6009 (*National Instruments, Austin, Texas, USA*) data acquisition system was connected to the Millar PCU-2000 Pressure Control Unit through ¼ inch phono-to-BNC cables. The pressure control unit sent analog voltage signals from the venous catheter to the data acquisition device, which in turn interfaced with LabVIEW (*National Instruments*) to record the pressure waveforms with a sampling frequency of 1000 Hz.

### FEMORAL ARTERIAL PRESSURE WAVEFORM

The femoral arterial (FA) pressure waveform was obtained by inserting an MLT0670 disposable blood pressure transducer (*ADInstruments, Colorado Springs CO, USA*) into the femoral artery of the left leg. The pressure transducer was connected to an FE221 bridge amplifier (*ADInstruments*) by MLAC11 Grass adapter cables, which was then connected to a USB-6009 data acquisition system (*National Instruments*) by BNC-to-BNC cables. The USB-6009 data acquisition system then interfaced with LabVIEW (*National Instruments*) which recorded the FA waveform with a sampling frequency of 1000 Hz.

### VARYING ANESTHETIC AND HEMORRHAGE STATUSES

Once the catheters were placed, each animal underwent a 10-minute stabilization period. The inhaled isoflurane anesthetic was increased to a minimum alveolar concentration (MAC) of 1.8% (MAC 1). The subject underwent a 10-minute stabilization period at the MAC 1 concentrations, then the pressure waveforms were collected over a 15-minute period. This process was repeated for two additional MAC levels of 2.5% (MAC 2) and 2.8% (MAC 3). After the final data collection period for inhaled isoflurane, each subject was gradually reduced to a MAC level of 1.5% and underwent a 10-minute stabilization period prior to the increasing levels of infused propofol. The same procedure was followed for three varying levels of infused propofol: 0.1 mg/kg/min (Propofol 1), 0.15 mg/kg/min (Propofol 2), and 0.2 mg/kg/min (Propofol 3). After the final data collection window for infused propofol, each subject was gradually reduced to a 0.5 mg/kg/min baseline of propofol. Each subject underwent a 15-minute data collection period during stabilization to account for the euvolemic baseline (Before Bleeding). The subject was then exsanguinated via venous draw into evacuated bags that were continuously weighed until each subject had hemorrhaged approximately twenty percent of its blood volume. The volume of blood removed from each subject is displayed in Table 1.

The pressure waveforms were continuously collected over the 15-minute hemorrhage protocol (During Bleeding). Finally, after exsanguination was complete, a final 15-minute data collection period during stabilization was recorded to account for the hypovolemic baseline (After Bleeding).

### SPECTRUM ANALYSIS

The venous and FA waveforms collected in LabVIEW were exported to MATLAB *(MathWorks Inc.*) for data analysis. The pressure waveforms were passed through a lowpass filter with a normalized bandpass frequency of 15 Hz. Previous studies have suggested that because of the low amplitude nature of peripheral venous pressure waveforms, segments within the range of 0 to 20 Hz are ideal for processing ^19^. Thus, 15 Hz was deemed an appropriate setting for the lowpass filter. After filtering the data, the venous and FA waveforms were sectioned into 20 second and 30 second snippets, then, a Fast Fourier Transform (FFT) and power spectral density (PSD) were performed on the snippet using the following equations:

$$Y(k)=\sum_{j=1}^{n}\;X(j)W_{n}^{\left(j-1)(k-1\right)}$$

$$X(j)=\frac{1}{n}\sum_{k=1}^{n}\;Y(j)W_{n}^{-(j-1)(k-1)}$$

$$\mathrm{Power}\;=\frac{|Y{|}^{2}}{n}$$

**Figure 1:**
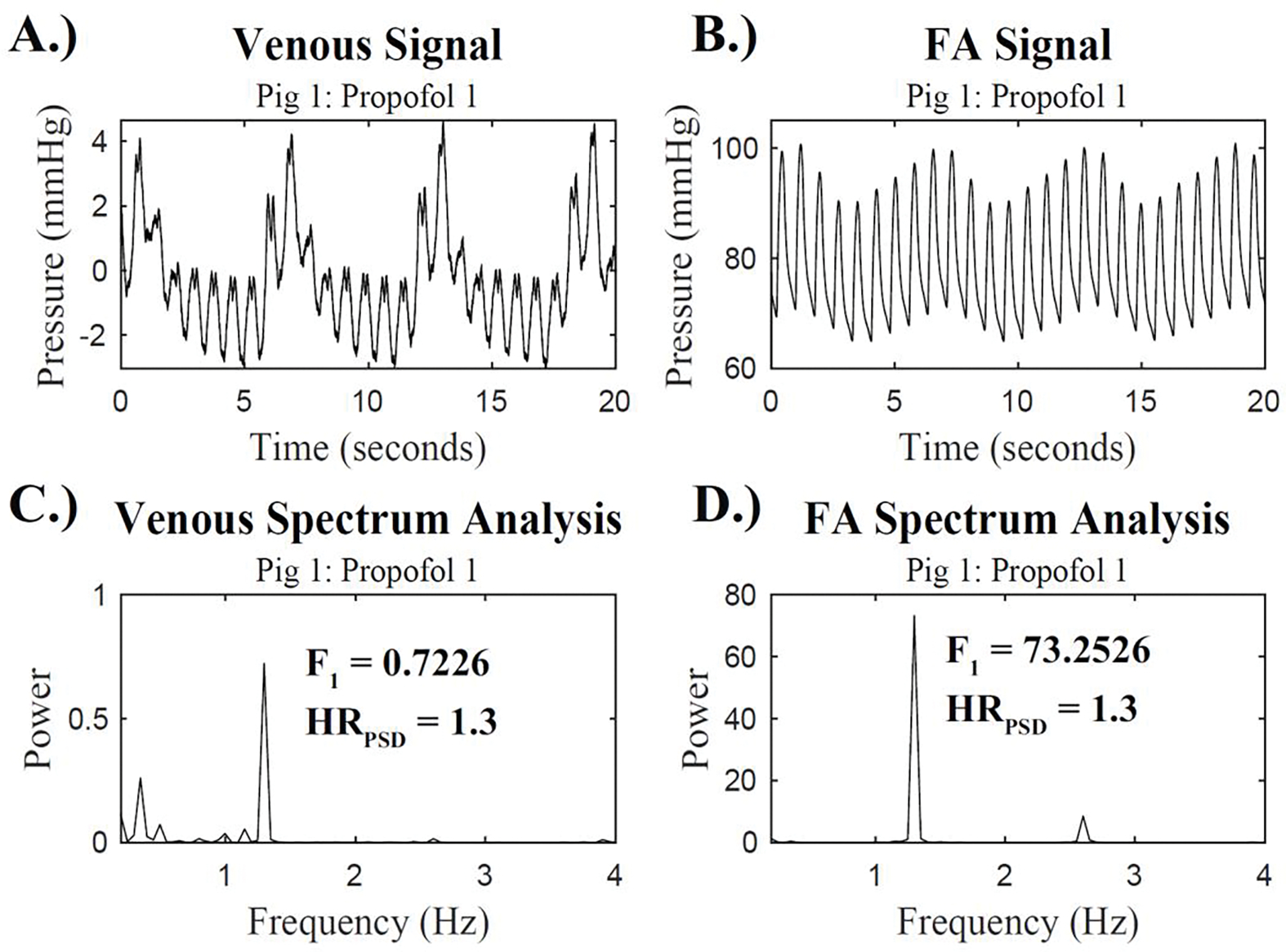
Time Domain and Spectrum Analysis Waveforms: A.) The venous pressure waveform and B.) the simultaneous FA pressure waveform for porcine subject 1 during Propofol 1. The corresponding power spectral densities are displayed for C.) the venous pressure waveform and D.) the FA pressure waveform with the F_1_ power and HR_PSD_ displayed.

The maximal power in the heart rate range was determined. A range between 0.5 Hz (30 bpm) and 3.5 Hz (210 bpm) was used to account for physiologically relevant heart rate frequencies ^21^. This power was labeled as the power at the heart rate frequency, F_1_ power. The frequency that this maximal power occurs at was identified as the heart rate frequency, HR_PSD_. This process was repeated to determine the F_1_ power and the HR_PSD_ for all 20 and 30 second snippets. Figure 1 demonstrates the time domain waveform and the respective PSD of venous and FA waveforms for a 20-second snippet with the F_1_ power and HR_PSD_ labeled.

### INSTANTANEOUS HEART RATE DETERMINATION

**Figure 2:**
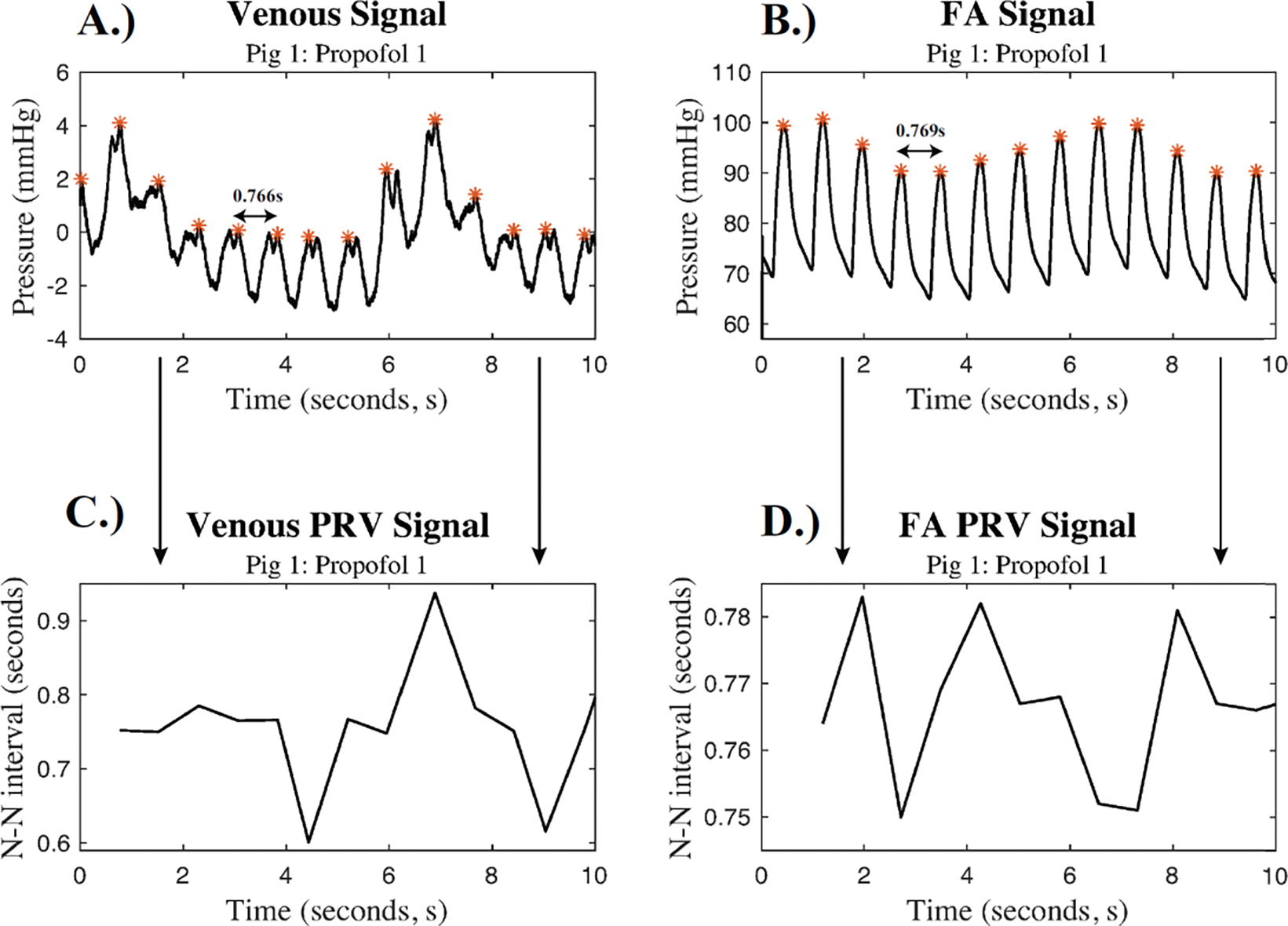
Signal Extraction Waveforms: A.) Filtered venous pressure waveform with N-N intervals marked and B.) the simultaneously filtered FA pressure waveform with N-N intervals marked for porcine subject 1 during Propofol 1. An orange (*) denotes pulses in the pressure waveforms. The corresponding pulse rate variability signals for C.) the venous signal and D.) the FA signal.

From each 15-minute recording, pulse intervals were extracted from the time domain of the venous and FA signals using a beat detection algorithm. Each dataset was then normalized to obtain the N-N intervals, by passing the signals through a filter that excluded any beat-to-beat intervals smaller than 0.2 (arrythmias) or were 20% different than the previous valid RR-interval. Figure 2 represents the methods for extracting the pulse intervals and the corresponding N-N intervals for both the venous and FA signals.

The N-N signal generated from the FA waveform had less variance than the N-N signal generated from the venous waveform, so all further analysis utilized the FA waveform to determine the instantaneous heart rate. The N-N signals were then sectioned into snippets that corresponded to the 20-second and 30-second snippets used for the spectral analysis. The mean N-N intervals, HR_inst_, were calculated for each 20-second and 30-second snippet.

### CORRELATION COEFFICIENT

The difference between HR_inst_ and the HR_PSD_ derived from each 20-second snippet for both the venous and FA waveforms was calculated. Each of these differences was, then, correlated with the F_1_ power for the corresponding snippet using Pearson’s correlation coefficients, depicted in the following equation:

$$p(X,Y)=\frac{1}{N\;-\;1}\sum_{i=1}^{N}\;\left(\frac{X_{i}\;-\;\mu_{X}}{\sigma_{X}}\right)\left(\frac{Y_{i}\;-\;\mu_{Y}}{\sigma_{Y}}\right)$$

where X is the difference between HR_PSD_ and HR_inst_, Y is the F_1_ power for the signal of choice, $\mu_{X}$ is the mean of $X,\;\sigma_{X}$ is the standard deviation of $X,\;\mu_{Y}$ is the mean of Y, and $\sigma_{Y}$ is the standard deviation of Y.

## Results

**Figure 3:**
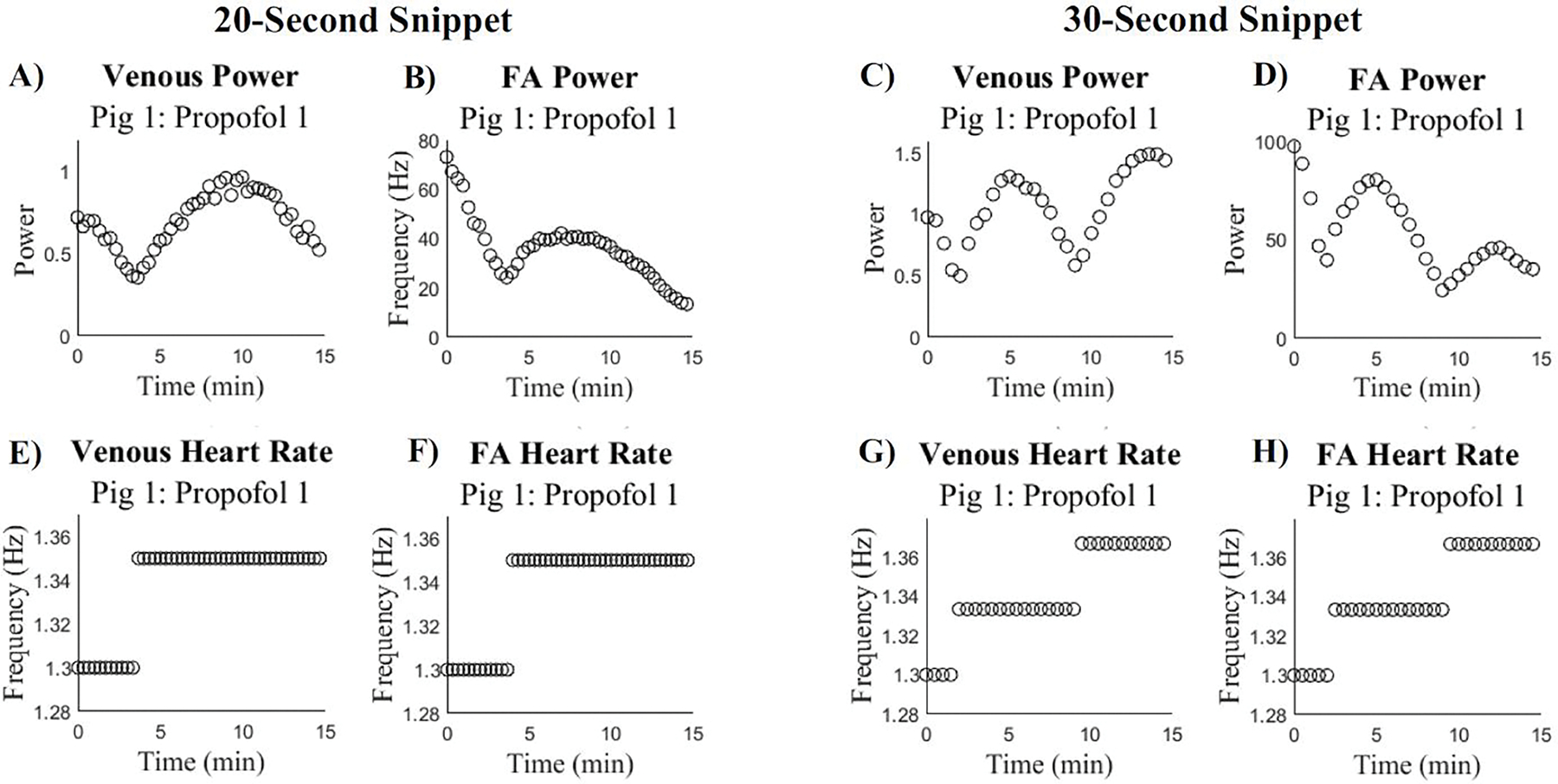
Power Spectrum Analysis of Subject 1 During Propofol 1 Using Different Snippet Sizes: A.) venous power, 20s snippets, B.) FA power, 20s snippets, C.) venous power, 30s snippets, and D.) FA power, 30s snippets. The corresponding heart rate for E.) venous power, 20s snippets, F.) FA power, 20s snippets, G.) venous power, 30s snippets, and H.) FA power, 30s snippets.

The spectrum analysis for both the venous and FA signals revealed non-linear trends in the F_1_ power. Analysis of the HR_PSD_ demonstrates that throughout the duration of the 15-minute signals, the average heart rate is changing. Additionally, it was found that there was a change in the trend seen in the F_1_ power for both the venous and FA signals that correspond to the change in HR_PSD_. These two trends are depicted in Figure 3 for both the 20-second and 30-second snippets.

**Figure 4:**
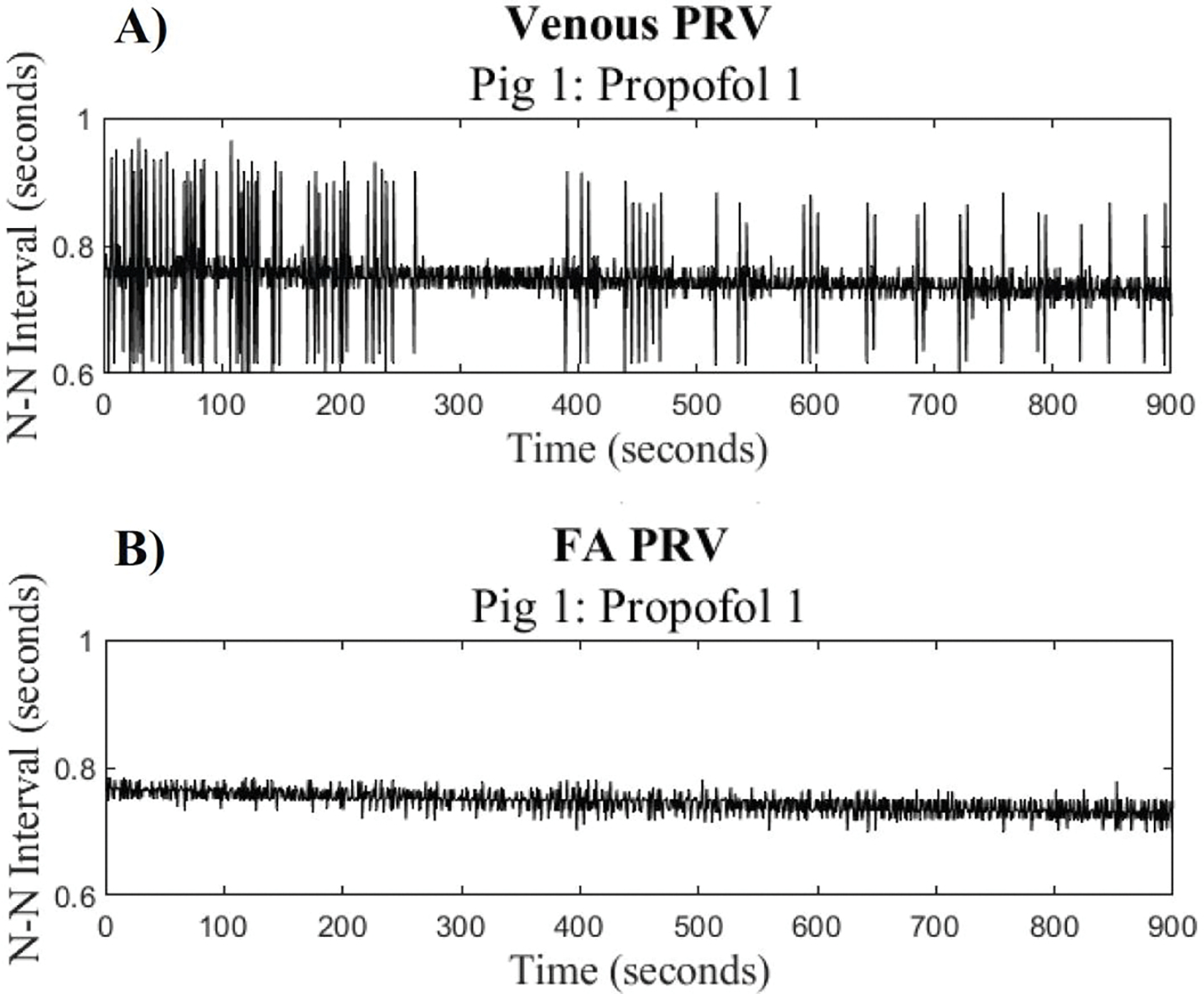
Comparison of HR_inst_ for Venous and FA signals: A.) The venous PRV (pulse rate variability) signal and B.) the FA (femoral artery) pulse rate variability signal for porcine subject 1 during Propofol 1.

To better understand the changing heart rate, we compared the trends in the HR_PSD_ with the HR_inst_ for both venous and FA signals. Figure 4 portrays the comparison between the HR_inst_ for the venous and FA signals.

The HR_inst_ derived from the FA signal shows less variance than the HR_inst_ derived from the venous signal. Therefore, all further analysis used the HR_inst_ derived from the FA waveform.

**Figure 5:**
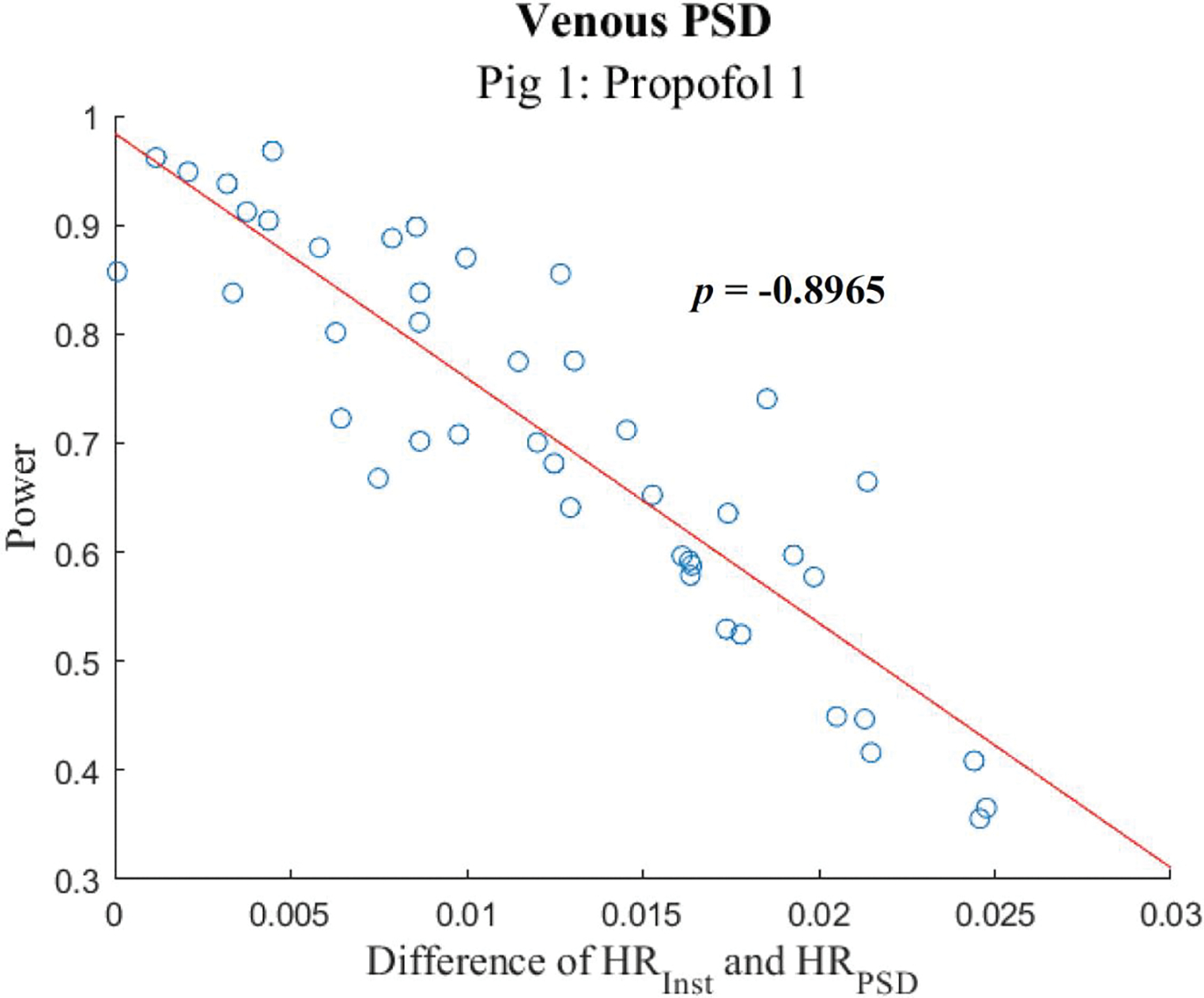
Subject 1 HR Correlation: Correlation plot comparing the differences between the mean instantaneous heart rate and the heart rate obtained from power spectral density with the F_1_ power obtained from 20-second snippets for porcine subject 1 during Propofol 1. The correlation coefficient for this data set is displayed. PSD: Power Spectral Density

For each snippet, the absolute difference between HR_inst_ and the HR_PSD_ from both the venous and FA waveforms was found. This difference was then correlated with the F_1_ power for that snippet. The correlation plot for the 20-second snippets during Propofol 1 is depicted in Figure 5.

The following tables display the correlation coefficients between the F_1_ power and the difference in heart rate frequencies for each 20-second snippet. Table 2 displays the correlation coefficients found between the F_1_ power and the differences in heart rate frequencies when using the spectral data from the 20-second snippets of the peripheral venous pressure waveform to determine the F_1_ power and HR_PSD_.

Table 3 represents the correlation coefficients between the F_1_ power and the difference in heart rate frequencies when using the F_1_ power and HR_PSD_ from the 20-second snippets of the FA waveform.

## Discussion

Most studies evaluating the utility of the venous and FA pressure waveforms through PSD and frequency domain analysis are studying portions or snippets of the signal ^13,22^. This increases the number of data points one can study but decreases the precision in the frequency domain. Frequency is measured in Hertz (Hz), which is equivalent to the inverse of seconds (s^−1^), so the precision in the frequency domain is controlled by the number of seconds in each snippet. For example, the 20-second snippets can only have a precision of 1/20 s^−1^ or 0.05 Hz. Thus, the average heart rate obtained from PSD analysis only appears in multiples of 0.05 Hz.

In Figure 3, there is a local minimum in the F_1_ power for both the venous and FA signals at the same time there is a change in the HR_PSD_. This trend is maintained across snippet sizes, but the change in average heart rate and the local minimum in F_1_ power appears at different times for the 20-second and 30-second snippets. We hypothesize that this phenomenon is a result of the different precisions in the frequency domain. For the 20-second snippet, the heart rate frequencies appear in multiples of 0.05 Hz and for the 30-second snippets, the heart rate frequencies appear in multiples of 0.033 Hz. When using 30-second snippets, there is greater precision in the frequency domain, which results in the average heart rate obtained from PSD analysis to be spread across more frequency values than the 20-second snippets. For example, if one signal has a range of heart rate frequencies between 1.30 Hz and 1.40 Hz, using 30-second snippets you would obtain heart rate frequencies at approximately 1.30, 1.33, 1.36, and 1.39 whereas the 20-second snippets would obtain heart rate frequencies at 1.30, 1.35 and 1.40. Thus, the HR_PSD_ changes at different times and the local minimum in F_1_ power occurs at different time points in the 20-second and 30-second snippets. The variation between the local F_1_ minimum and corresponding change in average heart rate between the 20-second and 30-second snippets suggests that the average heart rate is changing, and therefore the precision of that heart rate is the controlling factor in the changes in F_1_ power.

In PSD analysis, the strength of each frequency in a signal is shown. Therefore, a low power would suggest that there is a low presence of that frequency in the signal. Similarly, a high power would suggest that there is a high presence of that frequency in the signal ^23^. Additionally, if the heart rate is changing throughout a signal, the power at the heart rate frequency will be spread across multiple frequencies, but if the heart rate is constant, the power will be concentrated at that specific frequency and thus greater. We hypothesized that this phenomenon is contributing to the F_1_ power trends, as seen in Figure 3. As the true instantaneous heart rate approaches the average heart rate of the frequency domain, the F_1_ power would increase because the heart rate is more concentrated at the allowed frequency.

Since the snippet size influences the precision in the frequency domain and causes corresponding changes in the average heart rate obtained from PSD analysis, we decided to evaluate the trends in instantaneous heart rate due to the time domain’s constant precision, dictated by the sampling frequency. In this study, the sampling frequency was 1000 samples per second (chosen based on hardware requirements), so the instantaneous heart rate has a consistent precision of 0.001 Hz. To gain a further insight into how the heart rate may be influencing the changes in F_1_ power, we compared the instantaneous heart rate obtained from the time domain with the average heart rate obtained from PSD analysis. We found the difference in HR_inst_ and the HR_PSD_ from the 20-second snippets for both the venous and FA signals, then correlated this difference with the F_1_ power from the snippet to determine if the imprecision of the frequency domain was contributing to the strength of the F_1_ power. The correlation coefficients reported in Table 2 and 3 reveal a strong negative correlation for most of our data sets between the HR differences and the F_1_ power, which supported our hypothesis. This demonstrates that as the true instantaneous heart rate approaches the average heart rate allowed by the frequency domain, the F_1_ power increases, and as the difference between instantaneous heart rate and the average heart rate allowed by the frequency domain increases, the F_1_ power decreases.

It is important to note that a significant limitation of this study is the small sample size of porcine subjects. With a sample size of four subjects, we are unable to deduce broad generalizations from the results presented in this study. Future studies should include a larger sample size as a focus in order to strengthen the viability of results presented in this manuscript.

## Conclusion

Currently, researchers are utilizing FFT and PSD analysis to identify changes in the peripheral venous and peripheral arterial pressure waveforms that are associated with changes in blood volume. Many of these studies are proposing to use the heart rate frequency obtained from small segments of the pressure waveforms; however, in clinical applications, it is likely that the heart rate will be changing continuously as these signals are being taken. In this study, the changing F_1_ power was evaluated under varying snippet sizes for FFT and PSD analysis. In the frequency domain, the precision is derived from the number of snippets in the chosen segment. We found that the snippet-size chosen to analyze these signals can influence the trends in F_1_ power, which demonstrates that the precision available in the frequency domain has a strong influence on the spectrum analysis of the pressure waveforms. Additionally, the comparison of instantaneous heart rate with the heart rate obtained from PSD analysis suggests that the power at the heart rate frequency is indicative of the stability of the instantaneous heart rate. In future studies, these results should be validated by altering the heart rate of a subject while maintaining a consistent blood volume to determine if the power at the heart rate is being influenced by the changing heart rate or the changing blood volume.

## Figures and Tables

**Table 1:** Subject Blood Volume Removal: The volume of blood removed from each subject

Subject	Blood Volume Removed (mL)
Pig 1	1270
Pig 2	923
Pig 3	963
Pig 4	910

**Table 2:** Correlation Coefficients Comparing HR Frequency Differences with F_1_ Power: The correlation coefficients comparing the difference in heart rate frequencies with the F_1_ power. The power spectral density used to determine the power at the HR frequency and the HR obtained from the frequency domain was taken from the venous pressure waveform.

Porcine Subject 1				Porcine Subject 2			
	MAC	Propofol	Hemorrhage		MAC	Propofol	Hemorrhage
1	−0.9697	−0.8965	−0.9482	1	−0.6554	−0.8690	−0.9353
2	−0.9515	−0.8703	0.3878	2	−0.3304	−0.8660	0.053
3	−0.9027	−0.9365	−0.1136	3	−0.3675	−0.6593	−0.8959
Porcine Subject 3				Porcine Subject 4			
	MAC	Propofol	Hemorrhage		MAC	Propofol	Hemorrhage
1	−0.9032	−0.8764	−0.8898	1	−0.8841	−0.9500	−0.9693
2	−0.9874	−0.9001	−0.4676	2	−0.9081	−0.3619	0.5301
3	−0.7323	−0.8415	0.0910	3	−0.9450	−0.9715	−0.0401

**Table 3:** Correlation Coefficients Comparing HR Frequency Differences with FA Waveform F_1_ Power: The correlation coefficients comparing the difference in heart rate frequencies with the F_1_ power obtained from the FA pressure waveform. The PSD used to determine the power at the HR frequency and the HR obtained from the frequency domain was taken from the FA pressure waveform.

Porcine Subject 1				Porcine Subject 2			
	MAC	Propofol	Hemorrhage		MAC	Propofol	Hemorrhage
1	−0.9796	−0.6045	−0.9593	1	−0.9333	−0.9628	−0.9121
2	−0.8987	−0.7636	−0.2598	2	0.0631	0.1671	0.2168
3	−0.9521	−0.9717	−0.0433	3	−0.7764	−0.6129	−0.3132
Porcine Subject 3				Porcine Subject 4			
	MAC	Propofol	Hemorrhage		MAC	Propofol	Hemorrhage
1	−0.9220	−0.9598	−0.9701	1	−0.9931	−0.8400	−0.9798
2	−0.9834	−0.9578	−0.9543	2	−0.8742	−0.3073	0.8001
3	−0.9245	−0.9648	0.0884	3	−0.9511	−0.9761	0.0828

## Data Availability

The porcine datasets used in this study can be obtained by contacting the corresponding author.
